# Compression Creep and Thermal Ratcheting Behavior of High Density Polyethylene (HDPE)

**DOI:** 10.3390/polym10020156

**Published:** 2018-02-07

**Authors:** Rahul Palaniappan Kanthabhabha Jeya, Abdel-Hakim Bouzid

**Affiliations:** Department of Mechanical engineering, École de Technologie Supérieure, 1100 Notre Dame O, Montréal, QC H3C1K3, Canada; hakim.bouzid@etsmtl.ca

**Keywords:** compressive creep, thermal ratcheting, HDPE characterization, creep modulus, thermal ratcheting strain, bolted flange joints

## Abstract

The characterization of thermal ratcheting behavior of high density polyethylene (HDPE) material coupled with compressive creep is presented. The research explores the adverse influence of thermal cycling on HDPE material properties under the effect of compressive load, number of thermal cycles, creep time period, and thermal ratcheting temperature range. The compressive creep analysis of HDPE shows that the magnitude of creep strain increases with increase in magnitude of applied load and temperature, respectively. The creep strain value increased by 7 and 28 times between least and maximum applied temperature and load conditions, respectively. The creep modulus decreases with increase in compressive load and temperature conditions. The cumulative deformation is evident in the HDPE material, causing a reduction in the thickness of the sample under thermal ratcheting. The loss of thickness increases with increase in the number of thermal cycles, while showing no sign of saturation. The thermal ratcheting strain (TRS) is influenced dominantly by the applied load condition. In addition, the TRS decreases with increase in creep time period, which is cited to the extended damage induced due creep. The results highlight the need for improved design standard with inclusion of thermal ratcheting phenomenon for HDPE structures particularly HDPE bolted flange joint.

## 1. Introduction

In recent times, the polymer or plastic materials have seen a rapid growth in replacing the conventional metallic piping structures, mainly due to their economical production cost and minimal dependence and impact on the environment. The typical advantages of polymers over metallic materials are extensive protection against chemical and corrosion attacks, extended service life, and that they are lightweight with high strength and modulus. Amongst the different types of polymeric materials commercially available, high density polyethylene (HDPE) polymer has the second largest share of spoils behind polyvinyl chloride (PVC). HDPE is a good candidate for application in chemical fluid and slurry transfer pipes, because of its excellent chemical resistance and near frictionless flow characteristics. The applicability of HDPE material has seen a recent boom in the piping system against PVC, due to its excellent resistance to fatigue and UV radiation. The dominance of HDPE pipe in urban service piping network for water and gas, nuclear service water, and desalination piping, is evident. Similar to most polymer materials, the research on characterizing HDPE material properties is abundant. Since the early eighties, a large number of research studies have focused on the creep property of polymers, as this phenomenon is perceived as a hindrance and a drawback of polymer materials. Quantitative data on creep, and other perennial properties of two varieties of PVC and polyethylene materials under liquid pressure at different temperatures, was published [1]. The creep behavior of thermoplastics at temperatures close to the glass transition region of polymers was studied [2].

The viscoelastic creep response of high density polyethylene is explored in two creep models, a viscoplastic model and a nonlinear viscoelastic model, which on being fitted with experimental data, gave near perfect and moderate accuracy, respectively [3,4]. The developed nonlinear creep model of high density polyethylene has a good agreement with the experimental data, including the effect of ageing [5]. The recent enthusiasm towards viscoelastic property has lead into probing of the viscoelastic and viscoplastic behavior of HPDE under cyclic loading conditions [6]. Crack initiation and propagation of ductile and brittle polyethylene resin under creep damage show that only the brittle resin exhibits a lifespan controlled by slow crack growth (SCG) [7].

Researchers [8,9] studied the impact of strain rate and temperature on tensile properties of the post-consumer recycled HPDE. A large quantity of research focused on the mechanical cycling or fatigue behavior of HDPE material [10], HDPE geogrid [11], solid extruded HDPE [12], HDPE composite [13], HDPE pipe joints [14], but almost none on the thermal ratcheting effect. The mechanical property of filled HDPE and the effect of loading and manufacturing method on the properties of HPDE are well documented in the studies [15,16], respectively. Additionally, statistical analysis of HDPE fatigue life is performed [17]. The influence of cyclic loading rates under different temperature conditions on the cyclic creep behavior of polymers and polymer composites was examined [18]. The brittle and ductile failure under creep rupture testing of high density polyethylene pipes are thoroughly researched [19]. There are no typical references available on the creep data of polymeric bolted flange joint subjected to compression. In metallic bolted flange gasketed connections, the gasket component is usually blamed for relaxation, and hardly ever the flange material itself [20]. However, such is not the case with polymeric flanges, hence, quintessential analysis of compressive creep behavior is a necessity.

Furthermore, most polymer materials have restrictive features of low operational temperature conditions, thereby making them vulnerable to any temperature fluctuations. Consequences of thermal ratcheting or cycling of temperature on polymers can be severe, yet remain a relatively rare phenomenon in reported scientific literature. The work on the hot blowout testing procedure for polytetrafluoroethylene (PTFE) based gaskets [21], and the creep–thermal ratcheting analysis of PTFE based gaskets [22], are a few examples of the rarest research on the thermal ratcheting behavior of polymers.

In the present work, a detailed characterization of high density polyethylene under compressive creep, thermal ratcheting phenomenon, and the coupled effect of compressive creep and thermal ratcheting at different temperature and stress conditions, is presented. Even though the compressive creep problem of the polymeric bolted flange joint is the driving factor for this research, the output can be adapted to a wider range of polymer applications.

## 2. Materials and Methods

### 2.1. Experimental Setup

**Figure 1:**
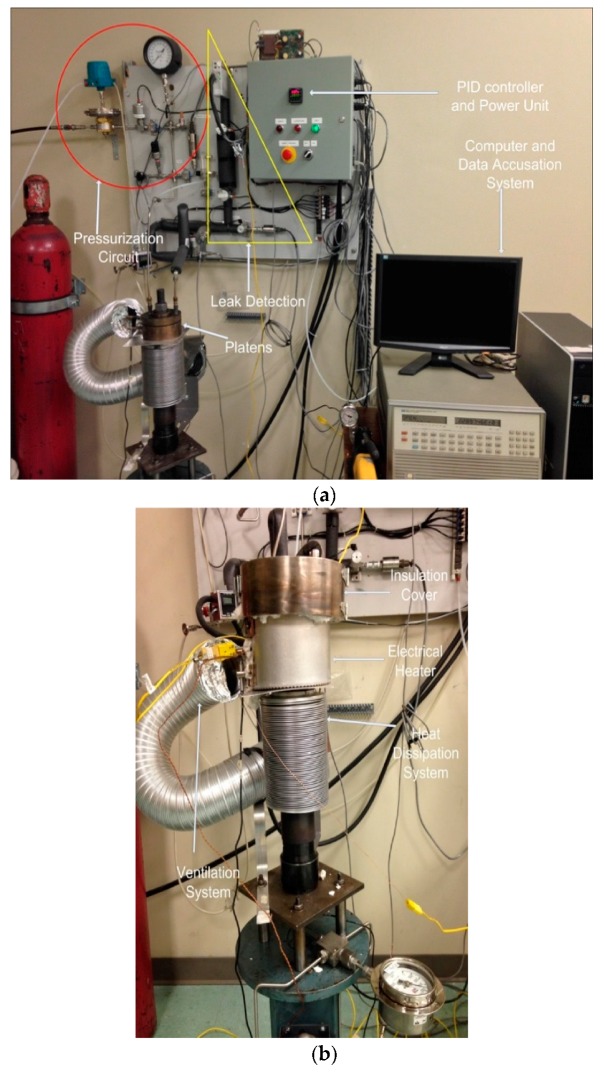
Universal gasket rig: (**a**) entire unit; and (**b**) heating system.

The universal gasket rig (UGR) is an innovative in-house built experimental test bench for performing mechanical and leak characterization of polymeric materials, shown in Figure 1a. The significance of UGR is highlighted through the capacity to perform intricate compressive creep and thermal ratcheting analysis of HDPE material. Conceptually, the UGR generates a simple distributed compressive load on the specimen with hydraulic pump and two platens. A central stud, screwed to the hydraulic tensioner head, transmits the required compressive stress to the sample. The conservation of load on the material is achieved through an accumulator connected with hydraulic system. The UGR can facilitate ring shaped samples with a maximum outer and minimum inner diameter of 100 and 50 mm in between the two platens. The polymer test pieces are limited to an allowable thickness of 10 mm. This simple and sophisticated machine supports the complex analysis of material properties through the ability to apply an integrated load of internal pressure, compression, and heat. Typically, the maximum operating condition of UGR unit is restricted to 5 MPa of internal pressure on a controlled high temperature environment of 450 °C.

The real-time reduction in the thickness of the sample under compressive creep is measured using a high sensitive linearly variable differential transformer (LVDT). The samples are heated by means of an external ceramic band, which encloses around the platens to apply heat on the sample in form of conduction (illustrated in Figure 1b). A proportional integral derivative (PID) controller is used to control the temperature of the heater by monitoring the temperature of gasket through a thermocouple, which is connected to a computer by RS232 serial port. The rate of heating is set at 1.5 °C/min, while the cooling is accomplished through natural convection by shut-off of the heater once the desired temperature is attained. The rigidity is controlled using an appropriate number of Belleville washers.

A special insulation cover, comprising fiber materials and a stainless-steel cap, successfully accomplishes the prevention of heat loss to the surroundings. A Wheatstone bridge circuit strain gauge is affixed at the bottom of the central stud, to measure the strain induced on the test piece due to the application of load. The machine can apply a maximum stress of 70 MPa on a sample area of 645.16 mm^2^. Specially designed inlet and outlet ports in the upper platens are handy in pressurizing the internal surface and measuring the leak rate under different test conditions. The exclusive feature of UGR is the thermal ratcheting analysis, which is accomplished by the combined use of PID controller and LabVIEW program. The system requires a complete definition of ratcheting temperature range, number of temperature cycles, and time period of hold-off between thermal cycles to achieve thermal ratcheting.

### 2.2. Test Procedure and Material Specifications

**Figure 2:**
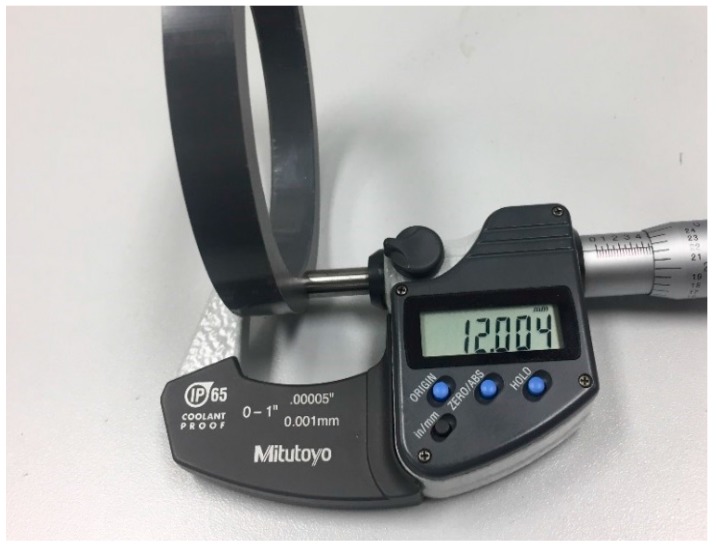
Specimen sample.

The mechanical characterization of ring shaped HDPE material is achieved through the sophistication of the UGR test bench. Typically, the physical measurement of polymer dimensions is the start point for the test procedure (Figure 2), followed by initialization of LabVIEW program to set up all the measuring sensors. The measured polymer ring dimensions are fed as inputs, which are used in the evaluation of applied compressive stress from the measured compressive strain by full bridge strain gauge. Initially, manual tightening of the nut is required to hold the ring specimen in position between two the contacting platen surfaces before any application of load through hydraulic pump. The zero position for LVDT sensor and sample gasket stress is defined at the instant of locking the platens manually. Subsequently, depending on the requisite of test to be performed, the chosen load level is exerted on to the polymer specimen, before or after the application of heat. In accordance with the industrial fluid process heating rate, the ceramic band electrical heater is set at a heating rate of 1.5 °C/min. Specially developed LabVIEW program provides for facile real-time monitoring of all the sensors of the test rig, while also enabling for options to modify the temperature and pressure conditions. The system has the capacity to monitor changes in the test conditions at a minimum interval of 10 s, with option to record values at a time interval between 10 to 600 s, as necessitated by behavior of the material over time. The creep and thermal ratcheting characterization of HDPE polymer is conducted in two phases. The first phase involves the short-term compressive creep analysis of the HDPE samples for 4 to 5 days under a variety of temperature and stress settings. The second phase is dedicated to the analysis of thermal ratcheting phenomenon, which is evaluated by preforming 10–20 thermal cycles between two target temperatures, with or without a day of creep. The information provided in Table 1 and Table 2 elaborates, in detail, the types of tests performed. The ring sample of HDPE material respects 3 inch iron pipe size (IPS), standardized to 72 mm and 90 mm as inner and outer diameter, along with a material thickness of 6.35 mm.

## 3. Results and Discussion

### 3.1. Creep Strain

**Figure 3:**
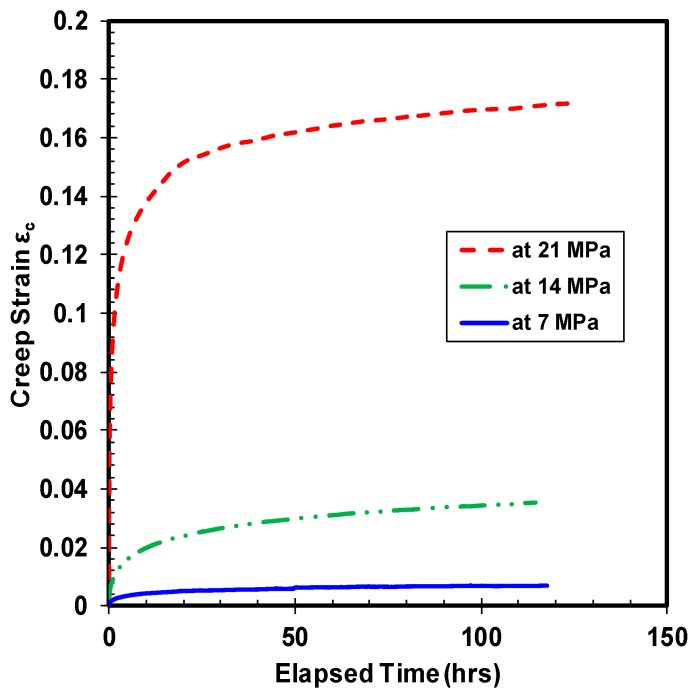
Creep strain under different loads at ambient temperature.

**Figure 4:**
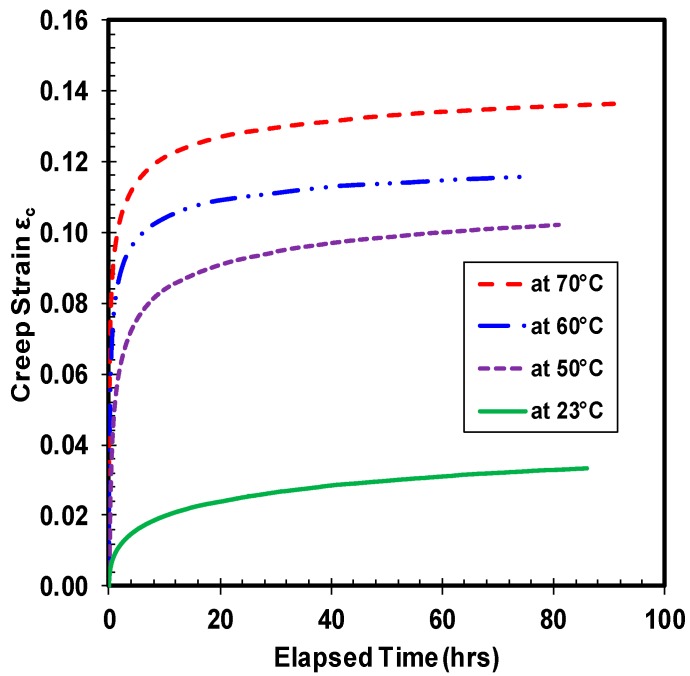
Creep strain under different temperatures at 14 MPa.

The vulnerability of polymer materials to creep and fatigue phenomenon are widely known, and high density polyethylene is no exception. Unlike metallic materials, the variation in creep strain of polymers under tensile and compressive load is distinct. Furthermore, the HDPE material is suspected to be prone to thermal ratcheting damage, due to inherent low melting temperature. Therefore, a quantitative assessment of thermal ratcheting behavior, coupled with compressive creep of HDPE material, is essential. The experimental creep test results highlight the importance of both applied compressive load and temperature on the creep strain of HDPE. Influence of applied load on creep strain is shown in Figure 3, where the magnitude of induced creep strain increases with increase in the value of applied compressive stress. Importance of applied load on the transition time from primary to secondary creep stage is evident, as the specimen demonstrates different time periods to reach the secondary phase. Yet, all HDPE samples demonstrate secondary creep, mostly within the first few hours of test. At ambient temperature, the creep strain of HDPE at 14 MPa of stress grew by six times the value of creep strain at 7 MPa of compressive stress. Whereas, on comparing the creep strain of HDPE at 14 and 21 MPa of compressive stress, the sample demonstrates a growth of 4.7 times the creep strain value at the lower load. The jump in magnitude of creep strain between 7 and 21 MPa of load is quite significant, which is nearly 28 times the former. The magnitude of the primary creep strain tends to increase with increase in applied load, causing subtle variations in the transition from primary to secondary creep for all the tested HDPE polymer samples. Alike to applied load, on varying the sample temperature under the same load (Figure 4), the creep strain tends to rise with intensification of temperature. Roughly, there is 20 percent increase in the creep strain with 10 °C escalation of temperature for HDPE material under the tested load. Even though the material’s response to creep under the two types of loading is similar, their magnitude of deformation is straight out different. The magnitude of primary creep phase amplifies with increase in the sample temperature under the same compressive creep load. Analogous to compressive stress, the magnitude of primary creep strain is proportional to applied sample temperature. A compressive creep test at a sample temperature of 70 °C was performed to understand if there is any drastic change in the material response, as the particular temperature is above the standard maximum operating temperature. The consequent reaction of HDPE sample is consistent with rest of the test results.

### 3.2. Creep Modulus

**Figure 5:**
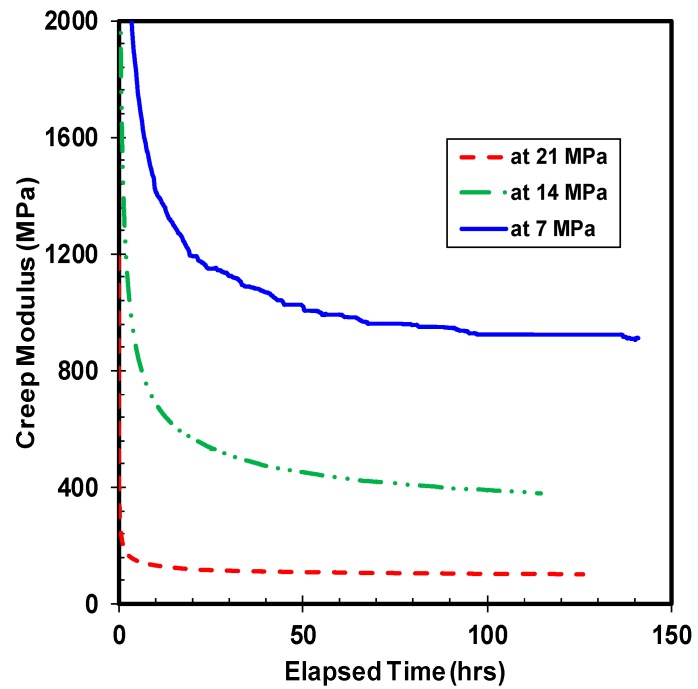
Creep modulus under different loads at ambient temperature.

**Figure 6:**
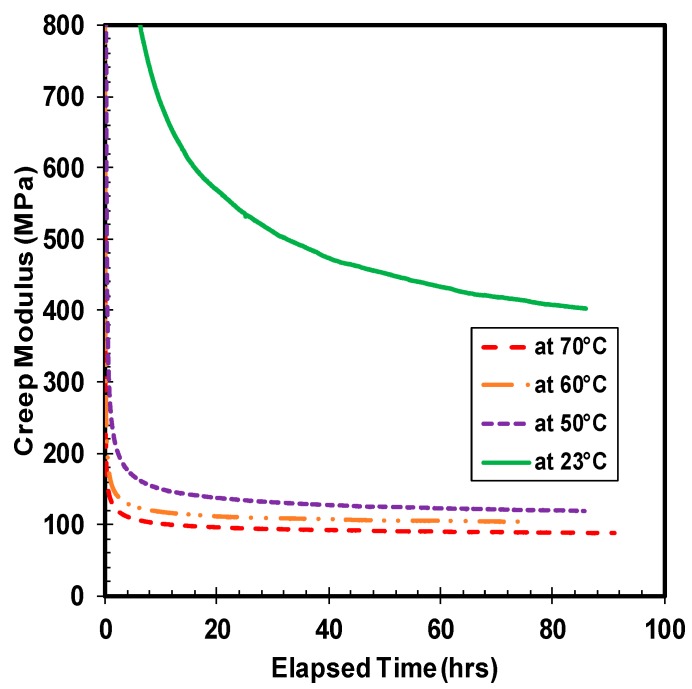
Creep modulus under different temperatures at 14 MPa.

Creep modulus is another important characteristic property of polymer material. The creep modulus is defined as the instantaneous elastic modulus of the material that varies with time. The ratio of creep stress over creep strain computes the creep modulus of the material. Since the creep stress is maintained constant, the creep modulus is inversely proportional to the creep strain. The creep modulus is dependent of the applied stress, where an increase of compressive load amplifies the loss in creep modulus of the material. The drop-in value of HDPE creep modulus over time under different compressive stress levels at room temperature is illustrated in Figure 5. The trend of creep modulus curve is similar to the creep strain curve, but in the inverse direction, where the material loses a substantial amount of creep modulus initially followed by a gradual saturation over time. The descent of creep modulus is maximum with the highest applied stress, while the magnitude of loss in creep modulus decreases as the value of applied compressive stress lowers. The saturation of creep modulus happens swiftly for the sample compressed under 21 MPa of stress, whereas the material requires a minimum of 10 and 30 h for transition to near saturation state under the compressive stress of 14 and 7 MPa, respectively. The change in creep modulus between 7 and 14 MPa of stress is higher than the difference in creep modulus between 14 and 21 MPa of stress, even though the increase in magnitude of compressive stress remains constant at 7 MPa. The probable reason cited for this behavior is the greater extent of irreversible damage induced in the specimen tested at higher compressive load, leading to earlier saturation. The influence of applied temperature on the creep modulus of HDPE is presented in Figure 6, which exhibits the loss of creep modulus with different chosen temperature under the applied compressive stress of 14 MPa. Consistent with the applied compressive stress condition, the trend in loss of creep modulus under different temperatures is similar where the highest reduction in creep modulus happens at the highest applied temperature under the same compressive stress. The magnitude of reduction in creep modulus between the room and high temperature samples is enormous. Besides, the saturation of creep modulus drop is evident with the highest temperature test, while the ambient temperature did not yield over the tested time period. The compressive creep test at 70 °C shows that the creep modulus drops by 8 times the initial value after saturation. By comparing the value of creep modulus at different temperatures, the consequence of temperature is apparent. At 70 °C, the material loses nearly 75% of the creep modulus value at 23 °C of temperature.

(1) $$\mathrm{Creep}\;\mathrm{Modulus}\;=\;\sigma_{c}/\varepsilon_{c}\;=\;\frac{Constant\;applied\;stress}{Creep\;strain\;at\;the\;instant},$$

### 3.3. Thermal Ratcheting

As mentioned earlier, the thermal ratcheting behavior is of utmost importance for polymeric materials, primarily due to their inherent property of low melting temperature. It is fair to say that a large selection of polymer materials can operate only in a moderate temperature conditions, hence, a small oscillation in temperature can cause a noteworthy change in the physical dimensions of the structure. Thermal cycling or thermal ratcheting induces a permanent cumulative deformation in the structure as a result of cycling of temperature under load. Similarly to mechanical ratcheting or fatigue, thermal ratcheting induces cumulative deformation on the material, where the scientific publications on this phenomenon is near to zilch. A proper understanding of thermal ratcheting phenomenon is important for HDPE bolted flange joint application, as a small loss in flange thickness would lead to a significant reduction in the bolt load, thereby causing a leakage failure. The test bench, universal gasket rig, facilitates the thermal ratcheting analysis of high density polyethylene material.

**Figure 7:**
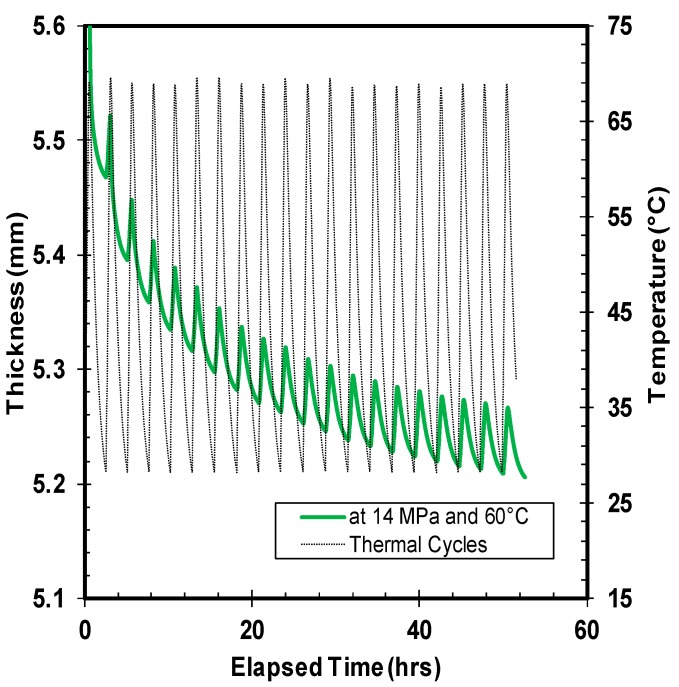
Thickness variation of high density polyethylene (HDPE) under 14 MPa of stress and a thermal ratcheting temperature range of 28 to 60 °C.

**Figure 8:**
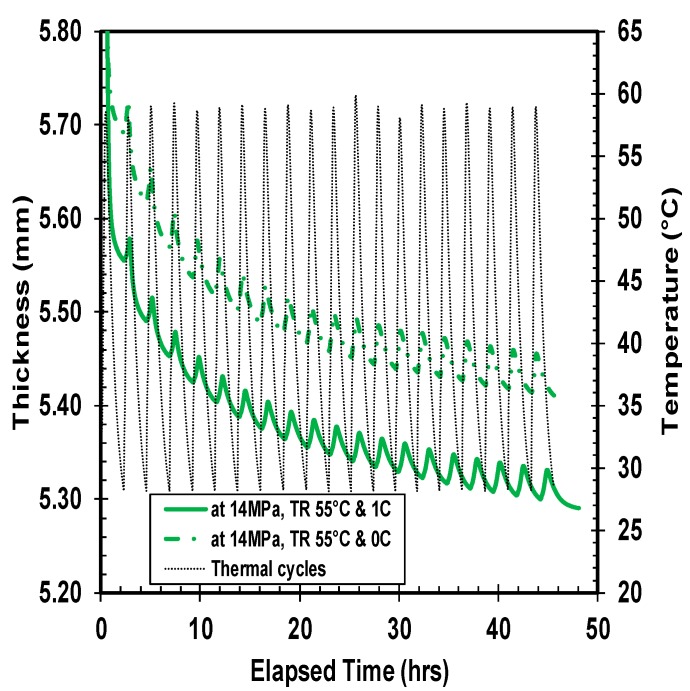
Ratcheting of HDPE with and without 1 day creep, at 14 MPa of stress.

**Figure 9:**
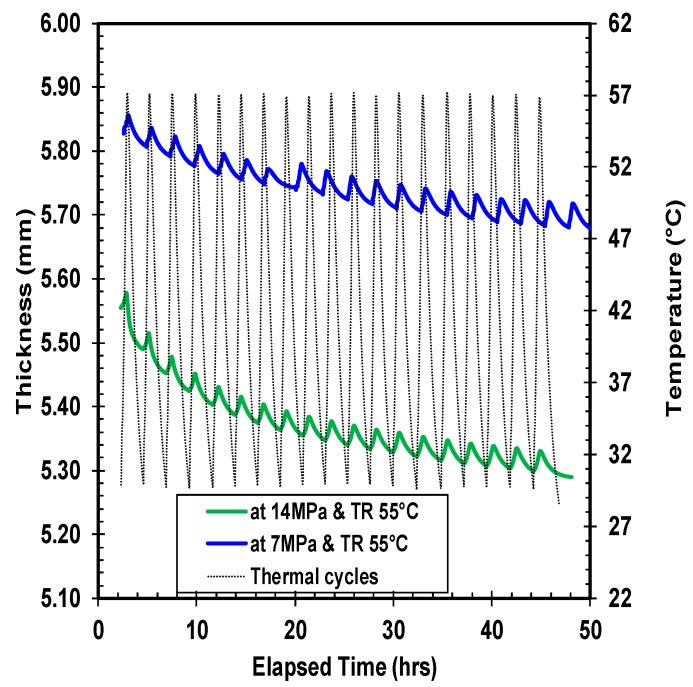
Ratcheting of HDPE under 7 and 14 MPa of stress.

Quantitatively, five different combinations of thermal ratcheting tests were performed to distinguish the consequence of creep time, applied load, and temperature on the thermal ratcheting strain of high density polyethylene. Under all thermal ratcheting tests, the cyclic escalation and reduction of thickness is apparent, with a net decrease in thickness. The wavy nature of the graphs (Figure 7, Figure 8 and Figure 9) illustrates the change in thickness under each thermal cycle. The rise and fall of thickness corresponds to the increase and decrease of temperature during the thermal cycling, which is clearly visualized with thermal cycles and thickness change plots in Figure 7. The material accumulates damage with each thermal cycle, causing an overall reduction in thickness. The significance of time period of creep and applied load on the thermal ratcheting strain is presented in Figure 8 and Figure 9, respectively. From the assessment of Figure 8, a substantial swift in thickness of HDPE samples tested at the same thermal ratcheting temperature, but with different initial period of creep, is noticeable. The amplification of the amount of deformation or reduction in thickness is partially cited to the initial creep of the material, while the rest is the coupled interaction of creep and thermal ratcheting, as the material simultaneously creeps and cumulates deformation, due to thermal ratcheting. On physical evaluation of the two-test specimen mentioned in Figure 8, the difference in thickness reduction is evident after removal of the applied load. Both samples demonstrated radial flow with increase and decrease of external and internal diameter, respectively. With the addition of one day of creep, an increase of 2% in the overall reduction of thickness is observed under similar ratcheting temperature. The impact of magnitude of applied compressive load on the thermal ratcheting deformation is demonstrated in Figure 9. HDPE samples tested under the same ratcheting temperatures, but with different applied stresses of 7 and 14 MPa, were evaluated to understand the importance of applied load condition. The specimen tested at 14 MPa of stress lost in excess of 7-fold of the thickness of the sample tested at 7 MPa after 20 thermal cycles. Since the two tests were performed without creep, the reduction in thickness is the coupled interaction of instantaneous compressive creep due to load and thermal ratcheting only. The cumulative deformation sustained during the initial cycles for HDPE material under 14 MPa is higher in comparison with the sample under 7 MPa of stress. This distinctive behavior is consistent with thermal cycles after 1 day of creep under 14 MPa, where the magnitude of initial damage, especially in the first four cycles, is great. A probable reason cited for this behavior is a change in the co-efficient of thermal expansion.

### 3.4. Thermal Ratcheting Strain

**Figure 10:**
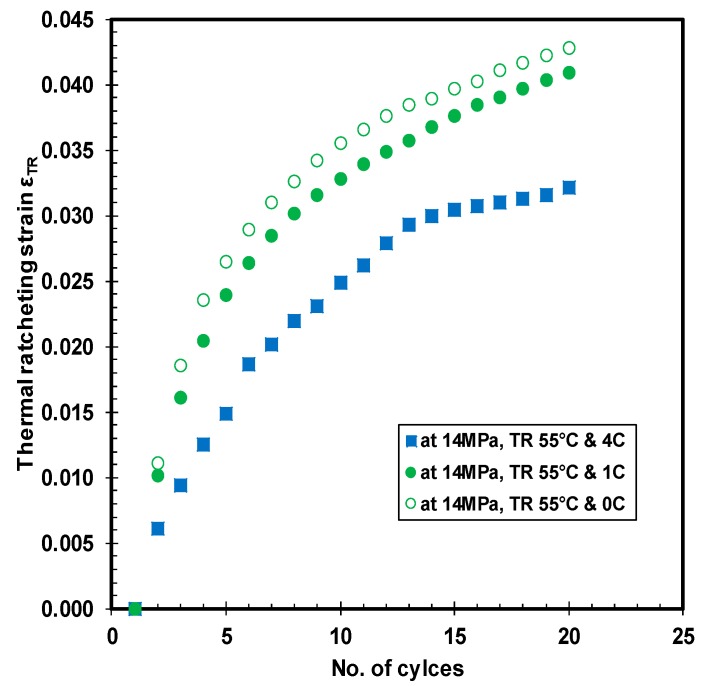
Thermal ratcheting strain under different time periods of initial creep at 14 MPa.

**Figure 11:**
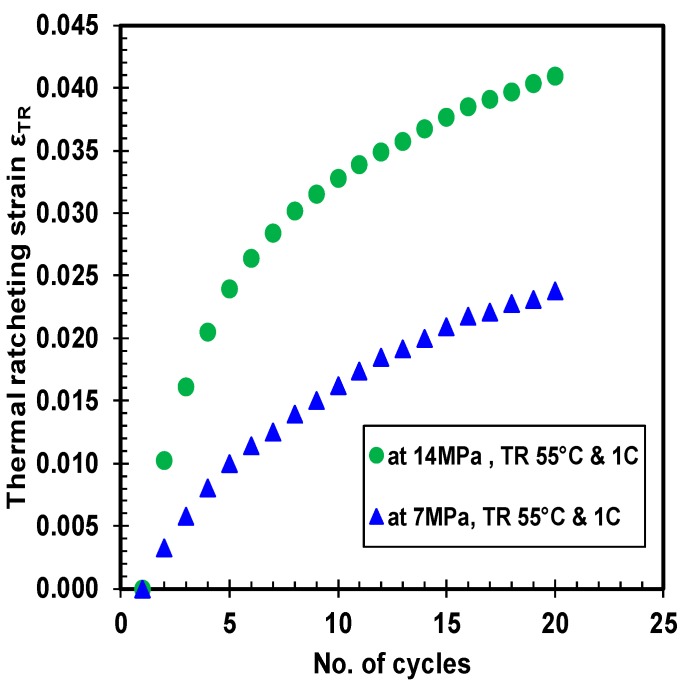
Thermal ratcheting strain at different applied loads.

**Figure 12:**
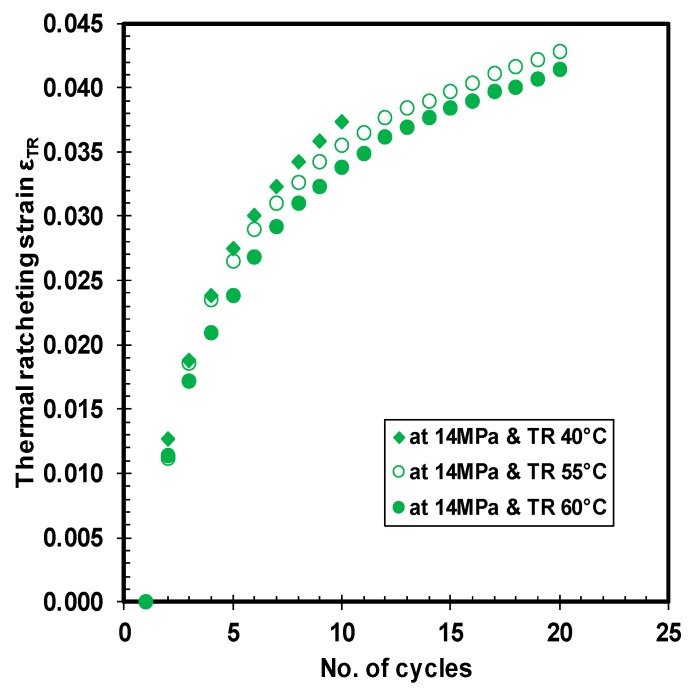
Thermal ratcheting strain under different ratcheting temperature ranges.

The thermal ratcheting strain (TRS) is the cumulative strain induced in the material as a consequence of thermal ratcheting phenomenon. The thermal ratcheting strain increases with increase in the number of cycles, which is observed in Figure 10, Figure 11 and Figure 12. The prominence of creep time period on thermal ratcheting strain is seen in the comparison between HDPE samples under the same cycling conditions, but at different initial creep time period. From the thermal ratcheting strain graph (Figure 10), it is noticeable that the TRS is higher for the sample tested without creep than the sample tested with one and four days of creep. This phenomenon indicates the effect of creep on the thermal ratcheting strain. Therefore, the creep time period is inversely proportional to the thermal ratcheting strain. Furthermore, the difference in thermal ratcheting strain between 1 day of creep, and without creep, is quite small, while maintaining a similar trend of thermal ratcheting strain. The thermal ratcheting strain increased by 10 percent for the test performed without 1 day of creep, in comparison to the test conducted with one day of creep. Moreover, the difference in TRS between 4 days of creep and zero days of creep is significant, where the TRS reduced by 36%, indicating that the effect of thermal ratcheting is severe without initial creep. The hardening of the material under creep is cited as the reason for increased resistance to thermal ratcheting, and consequently, reducing the magnitude of TRS with increase of creep time period. The vulnerability of HPDE to thermal cycling is undoubtedly exposed, prompting for further investigation. The change in TRS as an outcome of difference in applied compressive load is revealed in Figure 11. The variation in TRS between 7 and 14 MPa of stress is substantial, where the latter is higher than the former by approximately 1.9 times. It is noteworthy to mention that the TRS curve is much smoother at lower stress than at higher stress, where after the 7th thermal cycle, a change in slope of the curve is evident. Both the thermal ratcheting tests at the same ratcheting temperature range and different compressive stress, are executed after one day of creep.

Thermal Ratcheting Strain ε_TRSx_ = (LTR1 − LTRX)/Lo, (2)

where, LTR1 refers to the thickness of the sample after the first thermal cycle, while LTRX represents the thickness of the sample with each thermal cycle, and Lo is the initial thickness of the specimen.

Finally, the importance of variation in ratcheting temperature range while maintaining a constant load is demonstrated in Figure 12. The trend of the three thermal ratcheting strains is similar, with a slender variation in the magnitude. The tests were performed at three different ratcheting temperature ranges (28–40 °C, 28–55 °C, and 28–60 °C). Notably, the thermal ratcheting strain of the HDPE tends to decrease with an increase in ratcheting temperature range. During the initial few cycles, the difference in TRS for all the three ratcheting temperature ranges is minimal, but a clear deviation occurs after the 7th cycle of thermal ratcheting. Nevertheless, the thermal ratcheting strain, after 10 thermal cycles, was reduced only as little as 5% between 28–40 °C and 28–55 °C TR tests, while the difference is 4.7% between 28–55 °C and 28–60 °C tests. Unlike gasket materials [22], HDPE material did not saturate under 20 thermal cycles in the tested conditions.

## 4. Conclusions

The compressive creep and thermal ratcheting characterization of high density polyethylene (HDPE) material is successfully studied through universal gasket rig. The highlights of mechanical characterization tests are summarized:

- The HPDE material exhibits substantial creep damage under different compressive and thermal load conditions. The specimen shows a growth of 28 times the creep strain at 21 MPa from 7 MPa of compressive stress, while the increase in creep strain from the lowest tested temperature to highest tested temperature is 7-fold.
- The creep strain is directly proportional to the applied load and applied temperature, exposing the vulnerability of the material.
- Creep modulus is dependent on the applied stress. The magnitude of creep modulus decreases with increase in applied compressive load and material temperature. The maximum loss of creep modulus occurred at the highest applied stress and temperature, respectively.
- The impact of thermal ratcheting is evident, where the extent of cumulative deformation is dominated by the compressive load, followed by material temperature. The thermal ratcheting is very similar to the mechanical ratcheting or fatigue, causing an accumulation of deformation with each thermal cycle.
- In addition, all HPDE specimens demonstrate thinning of structural thickness under thermal ratcheting, and none of the specimens show any sign of saturation of deformation under the 20 tested thermal cycles.
- The thermal ratcheting strain of HDPE material is influenced by applied load, temperature, time period of creep, and number of thermal cycles. TRS is higher for the tests without creep, suggesting the deformation due to thermal ratcheting is critical during the initial period of the operation.

Finally, the coupled behavior of compressive creep and thermal ratcheting phenomenon of HDPE material raises the question on considering a common design criterion for polymeric materials, especially in bolted flange joint application. The results clearly indicate the necessity for upgraded design standards with inclusion of thermal ratcheting effect.

## Figures and Tables

**Table 1:** Creep test parameters.

High Density Polyethylene			
Test No.	Temperature (°C)	Stress (MPa)	Test Time Period
1	23	7, 14 & 21	5 days
2	50	7 & 14	5 days
3	60	7 & 14	5 days
4	70	7, 14 & 21	5 days

**Table 2:** Thermal ratcheting test conditions.

Test No.	Applied Stress (MPa)	Creep Temp (°C)	Ratcheting Temp (°C)	Days of Creep + No. of Thermal Cycles
High Density Polyethylene				
T1	7	23	28–55	1 + 20
T2	14	--	28–55	0 + 20
T3	14	23	28–55	1 + 20
T4	14	23	28–55	4 + 20
T5	14	--	28–60	0 + 20
T6	14	--	28–40	0 + 10
